# Significantly elevated serum human epididymis protein‐4 in chronic kidney disease patients without ovarian cancer: A large‐scale retrospective study

**DOI:** 10.1002/jcla.24847

**Published:** 2023-02-08

**Authors:** Shuidi Yan, Yong Lin, Xuelin Tian

**Affiliations:** ^1^ Clinical Laboratory, Zhongshan Hospital Xiamen University Xiamen China; ^2^ Department of Emergency, Zhongshan Hospital Xiamen University Xiamen China

**Keywords:** biomarker, chronic kidney disease, diagnostic performance, human epididymis protein‐4, ovarian cancer

## Abstract

**Backgrounds:**

Human epididymis protein‐4 (HE‐4) is a commonly used biomarker for diagnosing ovarian cancer. Elevated HE‐4 has also been observed in various benign conditions including chronic kidney disease (CKD); however, generalizability and statistical power of previous studies have been limited by small sample sizes.

**Materials and Methods:**

We conducted a retrospective study that included 80 pathologically confirmed ovarian cancer patients, 641 CKD patients, and 2661 healthy controls. Serum HE‐4 and several renal function parameters were collected and compared between the three groups. Correlation analysis was conducted to evaluate the relationship between HE‐4 and renal function parameters. A receiver operating characteristic curve was established to evaluate its diagnostic performance.

**Results:**

CKD patients had the highest levels of HE‐4, with a median of 193.00 pmol/L, while the median in ovarian cancer patients was 90.82 pmol/L. HE‐4 levels also increased with CKD progression, and Spearman's rank correlation showed that HE‐4 had a strong correlation with renal function parameters in CKD patients. Furthermore, HE‐4 exhibited a satisfactory diagnostic performance in both differentiating CKD patients and controls as well as stage 2 CKD patients and controls.

**Conclusion:**

HE‐4 can be used as an alternative biomarker for diagnosing CKD as it is less affected by several preanalytical factors. Nevertheless, in clinical practice, elevated HE‐4 requires taking both CKD and ovarian cancer into consideration.

## Graphical abstract

Elevated serum HE‐4 is common in CKD patients and positively correlated with the severity of CKD. It showed a strong correlation with certain renal function parameters in CKD patients and demonstrated a satisfactory diagnostic performance in both CKD patients and stage 2 CKD patients.

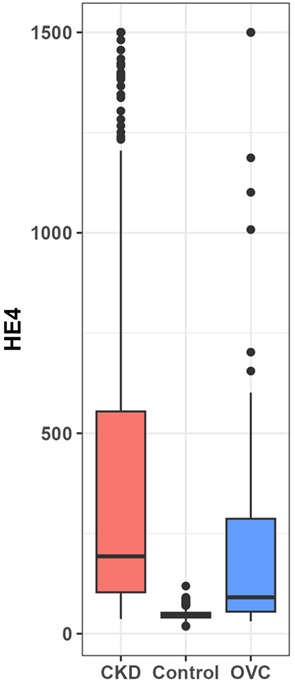

## 1 INTRODUCTION

Human epididymis protein‐4 (HE‐4) was first discovered by Kirchhoff et al.^1^ in 1991 and functions as a proteinase inhibitor.^1^ Initially, northern blots and in situ transcript hybridization indicated that HE4 mRNA specifically localized to the epithelial cells of the epididymal duct. Schummer et al.^2^ employed comparative hybridization of an array of 21,500 cDNAs in ovarian cancer and normal tissues, and the results showed that HE4 was highly expressed in cancer tissues, which demonstrated its potential as a diagnostic biomarker for this ovarian cancer.

Due to the rapid development of immunotechnology, HE‐4 level in serum can be measured using ELISA.^3^ This revolutionary discovery enables clinicians to order a HE‐4 test to help diagnose ovarian cancer. Combining HE‐4 with serum cancer antigen 125 (CA125) and the Risk of Malignancy Algorithm (ROMA) has become a power tool for diagnosing ovarian cancer and for distinguishing ovarian cancer from benign tumors.^4^ The establishment of an electrochemiluminescence immunoassay (ECLIA) for detecting HE‐4 along with fully automatic analyzers has greatly improved its diagnostic performance. The commercial ECLIA kit for HE‐4 has high sensitivity and precision, allowing the detection of trace levels of HE‐4, down to 15.0 pmol/L. Thus, measuring HE‐4 has become a common test in the medical examination among the female population, serving as a noninvasive cancer screening method. Additionally, HE‐4 has also been found to have diagnostic value in lung and other cancers. Taking lung cancer as an example, Cheng et al.^5^ conducted a meta‐analysis of seven individual studies and found an overall sensitivity of 0.72 and an overall specificity of 0.85 for HE‐4. Endometrial cancer, which is a common malignant tumor in the female population, is associated with significantly elevated HE‐4 expression in cancer tissues.^6^ Subsequently, a systematic review revealed that HE‐4 alone or combined with CA125 can be of critical importance for diagnosing endometrial cancer and for evaluating prognosis and survival.^7^

The wide utilization of HE‐4 promotes the early diagnosis and prevention of various cancers, especially ovarian cancer and endometrial cancer. That being said, using HE‐4 as a tumor biomarker in clinical practice sometimes causes confusion and misdiagnosis. An elevation of serum HE‐4 level, if not properly analyzed or interpreted, may lead to unnecessary consumption of medical resources. According to statistics released by Mills et al.,^8^ the global prevalence of chronic kidney disease (CKD) in men and women over 20 are 10.4% and 11.8%, respectively, which makes it a medical condition to not be neglected in the female population. Apart from regular dialysis and close monitoring of renal function parameters such as blood urea nitrogen (BUN), creatine (CREA), and cystatin C (CYSC), CKD patients are associated with a higher risk of certain cancers. Although ovarian, lung, and endometrial cancers were not significantly correlated with low estimated glomerular filtration rate (eGFR) in a large cohort study,^9^ clinicians often use proactive measures to screen tumor biomarkers in CKD patients, which means that CKD patients have a higher chance of taking cancer screening tests. Previous studies have reported elevated HE‐4 levels in CKD patients, with a median of 1041 pmol/L in women.^10^ This result was supported by Nagy et al.^11^ who conducted a comparison between controls and patients with different CKD stages. The results were consistent and showed that CKD stage was positively correlated with serum HE‐4 levels. Such approaches are unsatisfactory to some extent because the sample sizes were rather small. Although statistical significance was observed in previous studies, it is of critical importance to further clarify this topic with a larger sample size and explore the clinical significance as well as the possibility of HE‐4 serving as an alternative biomarker for evaluating renal function. Therefore, we conducted a large‐scale retrospective study to investigate associations among CKD patients without ovarian cancer and controls.

## 2 MATERIALS AND METHODS

### 2.1 Study participants

We conducted a retrospective study that included all consecutive adult female CKD patients treated at Zhongshan Hospital, Xiamen University (China) between 2017 and 2022. CKD patients were defined as individuals with incident CKD during the observation period using the Kidney Disease—Improving Global Outcomes (KDIGO) guidelines,^12^ with the first measurement of eGFR <60 ml/min/1.73 m^2^. The CKD‐Epi formula^13^ was employed to calculate the eGFR of CKD patients. Controls were age‐matched and randomly selected from people who underwent health examinations at our institution. Controls were excluded if they had eGFR <60 ml/min/1.73 m^2^ or the presence of ovarian cancer. Additionally, pathologically diagnosed ovarian cancer cases were enrolled to calculate the diagnostic performance of HE‐4.

### 2.2 Data collection

For each eligible study participant, clinical data including age, serum HE‐4 level, CREA, BUN, CYSC, and eGFR were collected and extracted from the laboratory information system. Biochemistry parameters were measured using a Beckman Coulter chemistry analyzer AU5800 (Beckman Coulter Inc.). HE‐4 levels were determined using a COBAS E602 immunology analyzer (Roche Diagnostics, Risch‐Rotkreuz, Switzerland) with ECLIA technology. The clinical data extraction was conducted by certified medical laboratory technicians, and all procedures were conducted in accordance with the manufacturers’ instructions and the Chinese National Guide to Clinical Laboratory Procedures.

### 2.3 Statistical analysis

Data were analyzed using SPSS 25.0 statistical software (IBM, Armonk, NY, USA). Quantitative variables with a normal distribution are expressed as mean ± standard deviation (SD) and were compared using the independent‐sample *t* test. If a quantitative variable did not conform with a normal distribution, the variable is expressed as median and interquartile range (IQR), and the Mann–Whitney U test was used to compare it between different groups. Linear regression was used to assess correlations between two quantitative variables with a Pearson's coefficient. A receiver operating characteristic (ROC) curve was established to analyze performance and to estimate the optimal cutoff value of certain biomarkers. A two‐tailed *p*‐value <.05 was considered statistically significant.

### 2.4 STATEMENT

This retrospective study extracted previous data from the laboratory information system, and no confidential information such as name, social security number, identity card number, or contact information were involved. The study design and procedures were approved and oversighted by the Ethical Committee of Zhongshan Hospital, Xiamen University. This study was carried out in accordance with the Declaration of Helsinki.

## 3 RESULTS

### 3.1 Study participants

In total, this study enrolled 80 pathologically confirmed ovarian cancer patients, 641 CKD patients, and 2661 healthy controls. The mean age (±SD) of the ovarian cancer patients, CKD patients, and healthy controls were 53.25 ± 12.35 years, 60.54 ± 16.32 years, and 56.20 ± 11.19 years, respectively.

### 3.2 Comparison of HE‐4 and renal function parameters between the three groups

**FIGURE 1:**
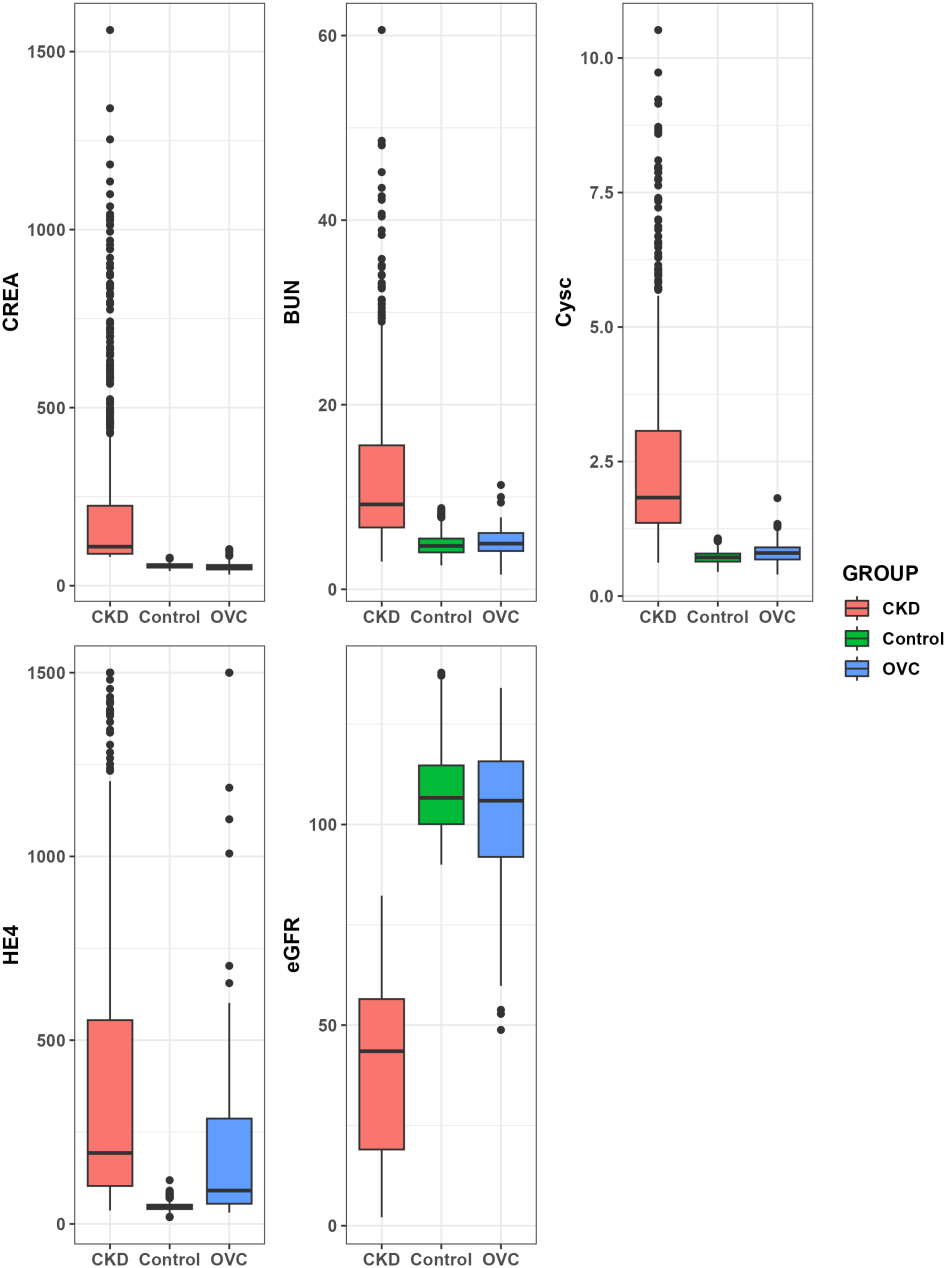
Serum HE‐4 levels and renal function parameters in the three groups

After data collection, we first examined the distribution of each parameter to determine the appropriate statistical method for subsequent analyses. As a result, HE‐4 and CREA were found to not conform with a normal distribution; therefore, these two parameters are presented as median (IQR) and a nonparametric test was used to compare differences between the groups. CysC, BUN, and eGFR were compared using one‐way ANOVA and the Bonferroni test for multiple comparisons. As shown in Table 1 and Figure 1, significant differences were observed in HE‐4, eGFR, and CREA between the three groups. Surprisingly, among the three groups, CKD patients had the highest HE‐4 levels, with a median of 193.00 pmol/L, while the median of the ovarian cancer patients was 90.82 pmol/L. CREA and eGFR exhibited similar trends in the three groups because eGFR is calculated using the CREA level. The control group had the highest eGFR and the lowest CREA levels. In contrast, the lowest eGFR and highest CREA levels were observed in CKD patients. In ovarian cancer patients, the levels of these two parameters were in between those of CKD patients and controls. Regarding BUN and CysC, we found no significant differences between ovarian cancer patients and controls, while CKD patients had significantly higher levels of BUN and CysC than ovarian cancer patients and controls.

**TABLE 1:** Comparisons of HE‐4 and renal function parameters between study participants

Parameters (units)	Ovarian cancer (*n* = 80)	CKD patients (*n* = 641)	Controls (*n* = 2661)	*p*‐value^a^	*p*‐value^b^	*p*‐value^c^
HE‐4	90.82 (237.45)	193.00 (452.45)	45.60 (12.01)	<.001*	<.001*	<.001*
eGFR	102.31 ± 18.69	39.01 ± 21.57	107.5 ± 9.84	<.001*	.001*	<.001*
CysC	0.83 ± 0.22	2.54 ± 1.81	0.72 ± 0.11	<.001*	.767	<.001*
BUN	5.15 ± 1.70	12.40 ± 8.44	4.83 ± 1.13	<.001*	1.000	<.001*
CREA	52.40 (13.73)	109.60 (138.00)	55.50 (9.70)	<.001*	.003	<.001*

*p < .05.

^a^ Ovarian cancer versus CKD patients.

^b^ Ovarian cancer versus controls.

^c^ CKD patients versus controls.

### 3.3 Comparison of HE‐4 and renal function parameters in CKD patients

**FIGURE 2:**
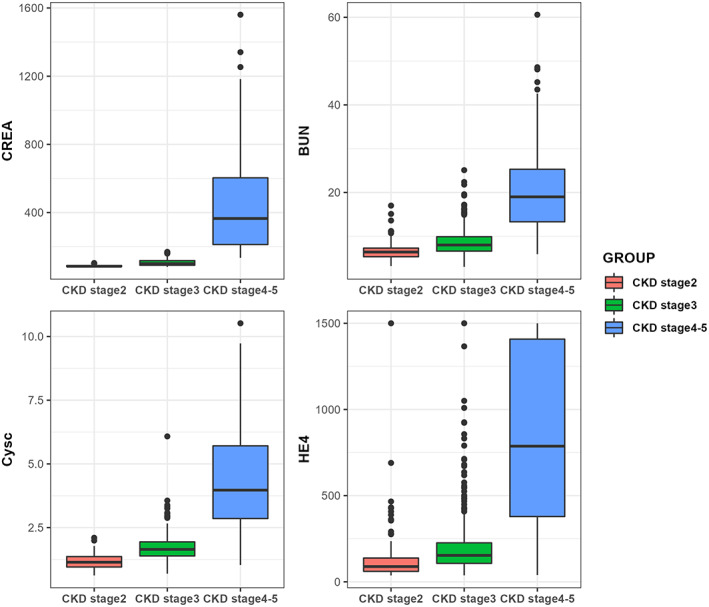
Serum HE‐4 levels and renal function parameters in CKD patients

We then further categorized the 641 CKD patients according to eGFR levels, and among them, 122 had stage 2 CKD, 296 had stage 3 CKD, 79 had stage 4 CKD, and 144 has stage 5 CKD. Given the limited number of stage 4 CKD patients, we combined the patients classified as stage 4 and 5 into a group for further statistical analyses. eGFR was ruled out in these comparisons because the CKD stages are based on it. After conducting the statistical analyses, we observed that HE‐4 levels were increased as CKD stages progressed, with statistical significance detected using a nonparametric test. CysC and BUN were also increased with CKD stages, and the levels of these two parameters were significantly higher compared with earlier CKD stages. However, we did not observe a significant difference in CREA levels between stage 2 and stage 3 CKD (*p* = .832). CREA levels were significantly higher when comparing stage 2 and stage 4–5 CKD, as well as stage 3 and stage 4–5 CKD. The detailed analyses are shown in Table 2 and Figure 2.

**TABLE 2:** Comparisons of HE‐4 and renal function parameters within CKD patients

Parameters (units)	CKD stage 2 (*n* = 122)	CKD stage 3 (*n* = 296)	CKD stage 4 and 5 (*n* = 223)	*p*‐value^a^	*p*‐value^b^	*p*‐value^c^
HE‐4	88.5 (78.73)	210.61 (119.95)	786.90 (1038.00)	<.001*	<.001*	<.001*
CysC	1.18 ± 0.28	1.71 ± 0.53	4.37 ± 1.93	<.001*	<.001*	<.001*
BUN	6.63 ± 2.18	8.75 ± 3.34	20.39 ± 9.36	.003*	<.001*	<.001*
CREA	86.38 ± 4.77	105.74 ± 19.02	444.08 ± 279.78	.832	<.001*	<.001*

*p < .05.

^a^ CKD stage 2 versus CKD stage 3.

^b^ CKD stage 2 versus CKD stage 4–5.

^c^ CKD stage 3 versus CKD stage 4–5.

### 3.4 Correlation analysis between HE‐4 and renal function parameters

**FIGURE 3:**
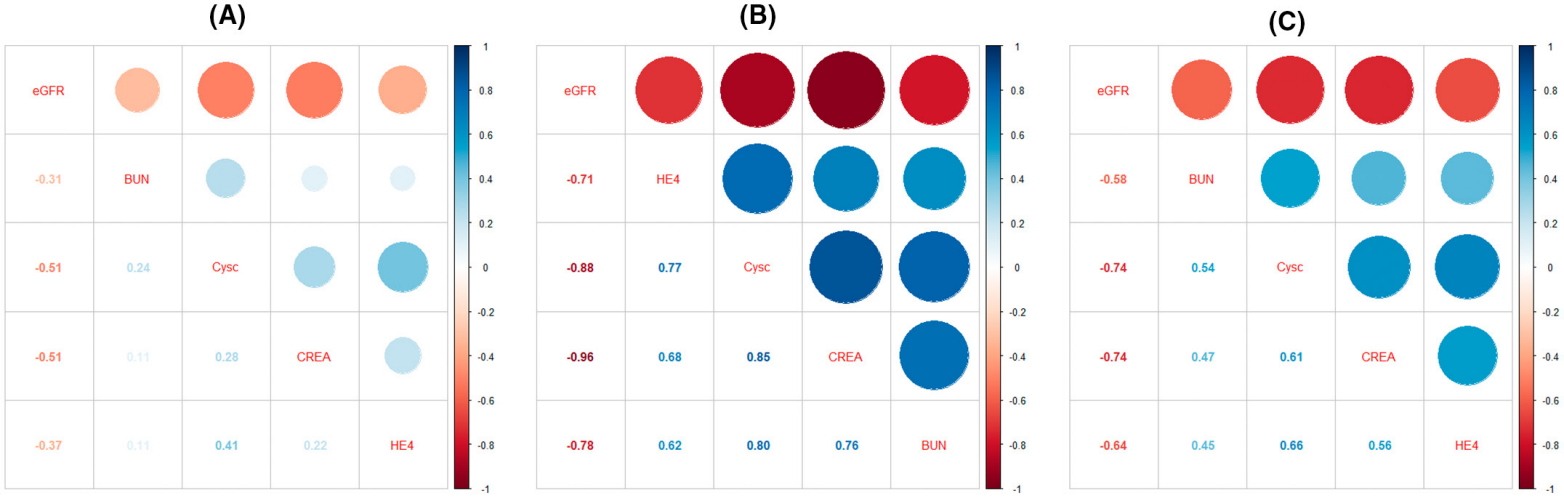
Correlation analysis of HE‐4 and renal function parameters in (A) controls (B) CKD patients, and (C) all study participants

As mentioned above, HE‐4 levels did not conform with a normal distribution; therefore, we employed Spearman's rank correlation to evaluate the relationship between HE‐4 and renal function parameters in CKD patients and controls. As shown in Table 3 and Figure 3, HE‐4 showed a significant correlation with renal function parameters, including BUN, CREA, eGFR, and CysC. Although statistical significance was observed in all groups, the ρ for each parameter was larger among CKD patients, indicating that the correlation was stronger in CKD patients. It was also noteworthy that the correlation in controls was rather weak, although significance was observed. Regarding parameters, CysC and eGFR had a stronger correlation with HE‐4 than BUN and CREA.

**TABLE 3:** Correlation analysis between HE‐4 and renal function parameters

Group	BUN	CREA	eGFR	CysC
CKD patients (n = 641)	0.621*	0.681*	−0.711*	0.772*
Controls (n = 2661)	0.105*	0.218*	−0.367*	0.409*
Total (n = 3302)	0.446*	0.562*	−0.644*	0.665*

*p < .05.

### 3.5 Diagnostic performance of HE‐4 in CKD

**FIGURE 4:**
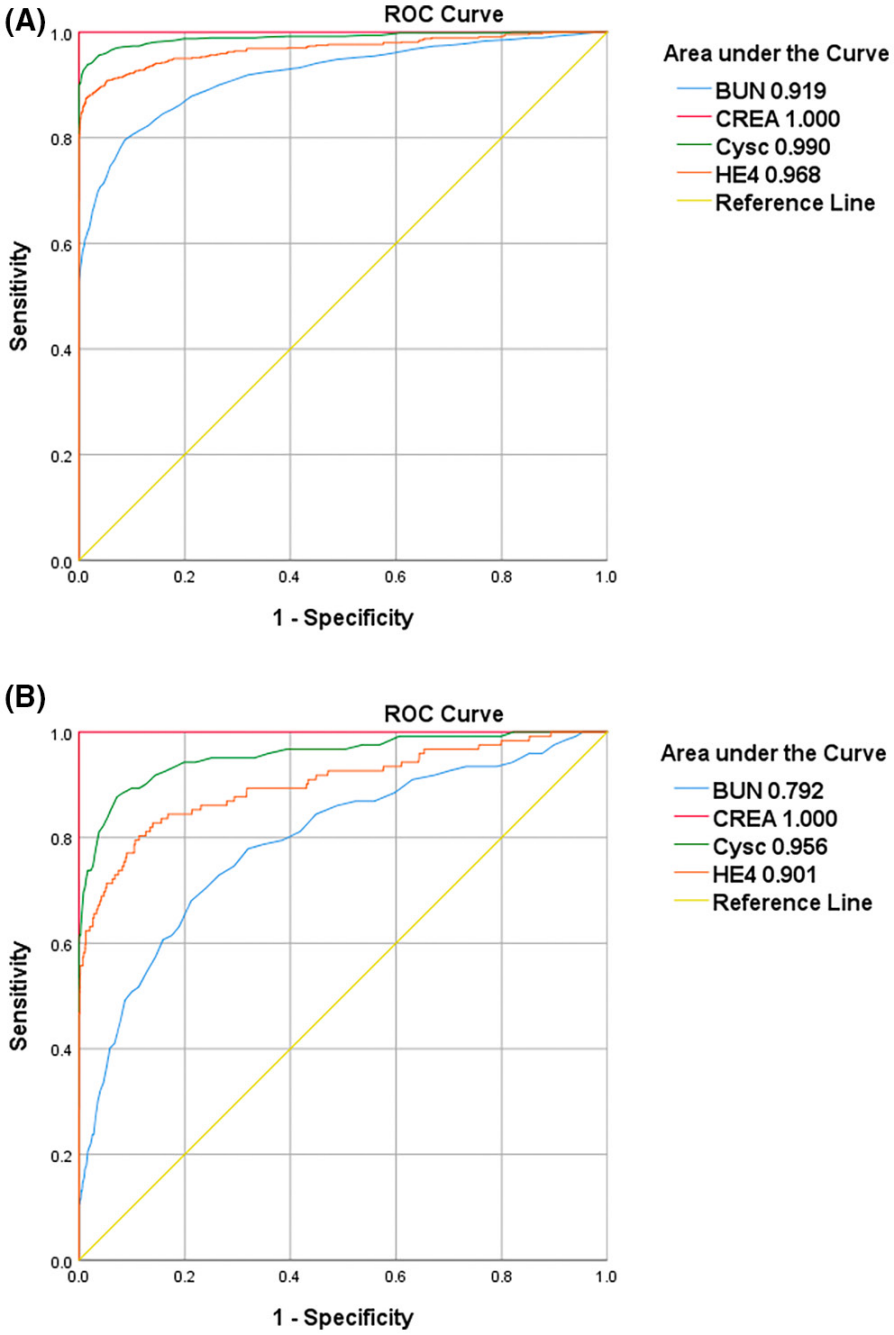
Receiver operating characteristic curve of (A) CKD patients versus controls and (B) stage 2 CKD patients versus controls

Because HE‐4 had a strong correlation with several renal function parameters, especially in CKD patients, we further evaluated its diagnostic performance for differentiating CKD patients and controls, as well as the associated renal function parameters. The ROC curves showed that CREA had the largest area under curve (AUC), and CysC also demonstrated a high diagnostic performance, with an AUC of 0.990. HE‐4, a previously acknowledged biomarker for ovarian cancer, had an AUC of 0.968, which is larger than that of BUN (0.919). We also attempted to test the diagnostic performance of HE‐4 and other renal function parameters for differentiating stage 2 CKD patients and control, and the results were largely similar to the above‐mentioned AUCs. HE‐4 had a high diagnostic performance, with an AUC of 0.901, which is higher than that of BUN (0.792) but lower than CysC (0.956) (Figure 4).

## 4 DISCUSSION

We conducted a retrospective study to investigate the HE‐4 level in ovarian cancer patients, CKD patients, and healthy controls with neither of these two conditions, as well as renal function parameters. We also compared HE‐4 levels in CKD patients according to their clinical stages. Moreover, we analyzed correlations between HE‐4 and renal function parameters in CKD patients and controls. HE‐4 showed good diagnostic performance for differentiating CKD patients and controls, as well as stage 2 CKD patients from controls, demonstrating its performance at diagnosing early‐stage CKD. Although prior studies^10^, ^11^ have noted elevated HE‐4 levels in CKD patients, the statistical power of these studies was limited due to small sample sizes. This study involved 641 CKD patients and 2661 controls to gain more robust results.

In this study, we observed higher HE‐4 levels among CKD patients, with a median of 193.00 pmol/L, than in ovarian cancer patients (90.82 pmol/L). The significantly elevated HE‐4 in CKD patients can be largely attributed to decreased clearance capacity caused by renal dysfunction, especially in end‐stage disease. This hypothesis was also validated by our analysis, as we found a linear trend in HE‐4 elevation as CKD progressed. As per our statistical analysis, the median HE‐4 level was 88.5 pmol/L in stage 2 CKD patients, reaching 210.61 pmol/L in stage 3 CKD patients and 786.90 pmol/L in stage 4 and 5 CKD patients, suggesting that eGFR is inversely correlated with HE‐4 level (ρ = −0.771). Apart from decreased clearance, renal fibrosis, which is a common pathological manifestation of renal damage, also can lead to elevated HE‐4 levels. Wan et al.^14^ reported an association between HE‐4 level and renal fibrosis score, with the elevation being significantly higher with IF/TA fibrosis grade. From the pathological perspective, LeBleu et al.^15^ explained the role of HE‐4 in the development of renal fibrosis and found that HE‐4 downregulated the activity of several kinds of proteases, including serine proteases and metalloproteinases, leading to an inhibition of their capacity to degrade type I collagen. Furthermore, the application of HE‐4 neutralizing antibodies can lead to the accelerated degradation of collagen I and inhibits fibrosis in an animal model. Taken together, these findings suggest that the elevated HE‐4 level in CKD patients is a manifestation of the combined effects of both upregulated expression in renal tissue due to fibrosis and decreased clearance capacity.

When we analyzed correlations between HE‐4 and renal function parameters, we found a stronger association between these parameters in CKD patients than in healthy controls. The correlation coefficients of HE‐4 were 0.621 (BUN), 0.681 (CREA), −0.711 (eGFR), and 0.772 (CysC) in CKD patients. In contrast, these were all reduced to a small level in controls, indicating that renal dysfunction is the source of these associations, and more importantly, that HE‐4 may not be useful as an alternative biomarker to evaluate renal function in a healthy population. The diagnostic performance of HE‐4 for differentiating CKD patients and controls was satisfactory, demonstrating an AUC of 0.963. The AUC of HE‐4 was lower than CREA and CysC but higher than that of BUN. This finding is consistent with that of Yuan et al.^16^ who evaluated the diagnostic performance of HE‐4 in 238 CKD patients and 230 controls.

It has been generally acknowledged that the clinical manifestations of CKD, such as anemia, nausea, itch skin, and high blood pressure are stealthy and common in other diseases and are mostly noticed by patients and clinicians when CKD progresses to advanced stages. An early diagnosis of CKD is of critical importance in preserving renal function, so we tested the diagnostic performance of HE‐4 in stage 2 CKD patients, which yielded an AUC of 0.901. This result reflects that elevated HE‐4 can be found in early‐stage CKD; thus, other laboratory tests of renal function should be taken into consideration if the possibility of ovarian cancer has been ruled out.

This study involved a large sample to evaluate HE‐4 as a biomarker for renal dysfunction, and detailed statistical analyses were conducted. Additionally, the comparison of renal function parameters between controls and CKD patients also suggested that the selection of study participants was appropriate and comparable. One of the limitations of this study is that the sample size of ovarian cancer patients was rather small; thus, it may not be an accurate estimation of the central tendency of HE‐4 in this group. Moreover, the application of renal biopsy is limited in clinical practice as it is an invasive procedure, so data on renal fibrosis in CKD patients are not available. As a result, an analysis of HE‐4 and renal fibrosis was not conducted in our study population. The absence of stage 1 CKD patients is another problem because they are rarely detected in clinical practice, so we could only evaluate the diagnostic performance of HE‐4 in stage 2 CKD patients.

This work revealed that elevated serum HE‐4 is common in CKD patients and positively correlated with the severity of CKD. Moreover, HE‐4 showed strong correlation with certain renal function parameters in CKD patients. It also demonstrated a satisfactory diagnostic performance in both CKD patients and stage 2 CKD patients. Our data suggest that HE‐4 can be used as an alternative biomarker in the evaluation of renal function, as HE‐4 measurement employing ELICA is more stable and is not affected by diet, muscle mass, and other preanalytical factors. Conversely, elevated HE‐4 also brings challenges for diagnosing ovarian cancer, as the prevalence of CKD is relatively high, especially among elderly people in the context of an aging society. Thus, HE‐4 results should be interpreted cautiously, and both CKD and ovarian cancer should be taken into consideration if elevated HE‐4 is found.

## CONFLICT OF INTEREST

None to declare.

## Data Availability

The dataset used in the present study is available from the corresponding author upon reasonable request.
